# Municipal and neighbourhood level wastewater surveillance and subtyping of an influenza virus outbreak

**DOI:** 10.1038/s41598-022-20076-z

**Published:** 2022-09-22

**Authors:** Elisabeth Mercier, Patrick M. D’Aoust, Ocean Thakali, Nada Hegazy, Jian-Jun Jia, Zhihao Zhang, Walaa Eid, Julio Plaza-Diaz, Md Pervez Kabir, Wanting Fang, Aaron Cowan, Sean E. Stephenson, Lakshmi Pisharody, Alex E. MacKenzie, Tyson E. Graber, Shen Wan, Robert Delatolla

**Affiliations:** 1 Department of Civil Engineering, University of Ottawa, Ottawa, K1N 6N5 Canada; 2 Children’s Hospital of Eastern Ontario Research Institute, Ottawa, K1H 8L1 Canada

**Keywords:** Civil engineering, Environmental sciences

## Abstract

Recurrent influenza epidemics and pandemic potential are significant risks to global health. Public health authorities use clinical surveillance to locate and monitor influenza and influenza-like cases and outbreaks to mitigate hospitalizations and deaths. Currently, global integration of clinical surveillance is the only reliable method for reporting influenza types and subtypes to warn of emergent pandemic strains. The utility of wastewater surveillance (WWS) during the COVID-19 pandemic as a less resource intensive replacement or complement for clinical surveillance has been predicated on analyzing viral fragments in wastewater. We show here that influenza virus targets are stable in wastewater and partitions favorably to the solids fraction. By quantifying, typing, and subtyping the virus in municipal wastewater and primary sludge during a community outbreak, we forecasted a citywide flu outbreak with a 17-day lead time and provided population-level viral subtyping in near real-time to show the feasibility of influenza virus WWS at the municipal and neighbourhood levels in near real time using minimal resources and infrastructure.

## Introduction

The World Health Organization (WHO) estimates that every year approximately 3,000,000–5,000,000 severe influenza infections occur, causing between 290,000 and 650,000 deaths globally^1^. In Canada, influenza is estimated to cause approximately 12,200 hospitalizations and 3500 deaths annually, making it one of the top 10 leading causes of death^1,2^. The significant impacts of seasonal and pandemic influenza on global health, particularly in the context of the potential deregulation of the traditional seasonality of respiratory pathogens after the onset of COVID-19, has created an urgent need for an improved surveillance of this disease. Standard clinical surveillance is resource intensive and is often a lagging indicator of community outbreaks. Furthermore, the data it generates is not sufficiently region-specific in terms of influenza activity and viral subtype^3^. Wastewater surveillance (WWS) may be used to test large populations for influenza using a single sample, similar to the application of SARS-CoV-2 WWS used to monitor the incidence of COVID-19 in communities^4–8^. WWS provides anonymous and aggregated data quickly at low cost and at potentially large scale through passive contributions by the community, and therefore may support and complement clinical surveillance programs and strengthen health emergency response systems in a similar manner to the tracking if the poliovirus during the twentieth century^8,9^. The potential of WWS as a public health tool therefore merits further development.

Influenza is a single-stranded RNA virus of the Orthomyxoviruses class and is divided into types A, B, C, and D. Influenza A virus (IAV) and influenza B virus (IBV) are typically associated with endemic seasonal influenza activity, and different subtypes of IAV are responsible for influenza pandemics^10,11^. The shedding characteristics of IAV and IBV suggests WWS could be used to identify and monitor seasonal influenza outbreaks and pandemics^12^. Previous studies have reported elevated fecal shedding rates of IAV and IBV of up to 10^6^ copies/g^13^. In addition to shedding the virus at high rates, infected individuals maintain fecal viral titers that are higher and persist longer than nasal viral titers^14,15^.

IAV and IBV are fecally shed at high rates, but since the virus has an outer lipid membrane envelop it is often presumed to be found in low concentration in wastewater. Hence the application of precise influenza WWS requires the optimization of methods to enrich influenza viral RNA from wastewaters. To do so, a thorough investigation of the partitioning of the influenza viral RNA signal in wastewater matrix is required. By elucidating the fraction of wastewater that influenza viral RNA signal is most prevalent, sample enrichment and concentration protocols can be optimized to yield higher viral signal recovery, and thus improve testing sensitivity and precision. The hydrophobic nature of the outer lipid membrane suggests that IAV and IBV should occur preferentially to the solids. In general, there has been limited success detecting endogenous IAV and IBV RNA in wastewater, and partitioning experiments have largely been limited to investigating spiked concentrations of viral surrogates in wastewaters^16–18^. The behavior of endogenous influenza virus RNA signal should differ from spiked viral surrogate within a wastewater matrix^19^. Preliminary investigations by Wolfe et al.^18^ hint that endogenous influenza virus RNA preferentially partitions to the solids fraction compared to polyethylene glycol (PEG) precipitated supernatant, which supports the hypothesis of disease target partitioning to the solids fraction of wastewater. As such, with the current knowledge of influenza partitioning in wastewater based predominantly on surrogate spiking experiments and on a single study of partitioning of endogenous IAV, additional study of endogenous influenza virus partitioning in wastewater is necessary to optimize the enrichment of disease targets and subsequently apply influenza WWS at the municipal and neighbourhood levels.

Numerous studies of influenza virus in natural water sources have analyzed the potential of oral influenza transmission pathways^17,20,21^, but only one has outlined a successful protocol for detecting endogenous IAV in municipal wastewaters^18^. The results demonstrated good agreement between wastewater measurements of IAV and reported clinical cases observed as part of the Michigan University’s campus surveillance program and Stanford University’s athlete surveillance program. However,there is insufficient information about detection and trend analysis of IAV WWS signals in cities and local communities, and most importantly, its relationship with clinical surveillance metrics. New information on IAV WWS is therefore required to bridge this gap. Furthermore, no study to date has demonstrated the potential for WWS to be used to subtype IAV. Subtyping could be especially useful to public health in identifying the presence of IAV strains responsible for community disease during seasonal epidemics. Because IAV may undergo antigen shift or drift, which favours pandemic-potential influenza features, it is important to develop methodologies to subtype IAV in wastewater. Even with the knowledge that 18 hemagglutinin (H1 to H18) and 11 neuraminidase (N1 to N11) subtypes exist for IAV, the subtypes that routinely cause human infections today are restricted to two strains, H1N1 and H3N2^11^. Identification of which subtype is responsible for community disease, with each subtype associated with distinct and clinically relevant phenotypic characteristics, would further increase the impact of IAV WWS.

In this study, we investigated the partitioning behaviour of endogenous IAV in municipal and neighbourhood wastewaters. We then used our findings to optimize an enrichment protocol for the quantification of influenza virus RNA in wastewater. Our optimized protocol was subsequently applied to quantify IAV and IBV RNA citywide wastewater as well as within three distinct neighbourhoods in Ottawa, Ontario, Canada. The wastewater viral signal was correlated with clinical case data available from Ottawa Public Health during an out-of-season outbreak to determine the benefits and complementary attributes of influenza virus WWS. Finally, IAV positive municipal and neighbourhood wastewaters were tested for H1N1 and H3N2 to identify the subtype that was responsible for community disease. This study is the first to show the applicability of influenza virus WWS and subtyping at the citywide and neighbourhood-levels during an influenza outbreak and the relationship between influenza virus WWS and subtyping to clinical surveillance.

## Results & discussion

### Partitioning of endogenous IAV virus in primary sludge and municipal wastewaters

Optimization of the enrichment and extraction method was conducted prior to initiating city and neighbourhood influenza WWS. The City of Ottawa and its neighbourhoods experienced an uncommon resurgence of IAV activity in the spring of 2022, which allowed for the collection of wastewater samples containing endogenous IAV. Sanger sequencing of PCR products confirmed the presence of IAV in samples from both WRRF and three neighbourhoods. Aligned sequences of the PCR products from primary sludge and municipal wastewater samples showed 92.5 and 98.1% homology with IAV (H3N2, Hong Kong/1/68/MA/E2 subtype), respectively. IBV was not detected in any of the clinical or wastewater samples. As such, only endogenous IAV fractionation experiments were performed on primary sludge (citywide samples) and municipal wastewater (neighbourhood samples), as endogenous IBV primary sludge and municipal wastewater samples were unavailable.

**Figure 1:**
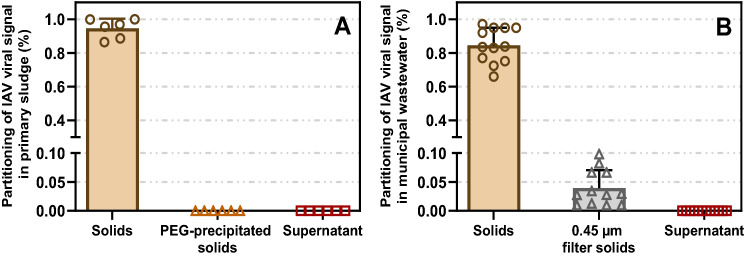
Partitioning of measured endogenous IAV viral signal present in: (**A**) primary sludge (n = 6); (**B**) municipal wastewater (n = 12). Means and standard error are displayed. Where the standard deviation is too small, the error bars are not displayed. Each measurement is based on three technical triplicates for each biological replicate.

In the primary sludge, IAV was found to almost exclusively partition in the solids fraction; analysis of primary sludge detected 88.1 ± 10.6% of the viral signal in the settleable solids with < 0.1% present in both PEG-precipitated solids and liquid fractions (Fig. 1A). Similar partitioning was observed in municipal wastewater; 84.6 ± 10.3% of the viral signal was in the settleable solids, 4.0 ± 3.1% was in suspended solids larger than 0.45 µm (filtered), and < 0.1% was in the supernatant (Fig. 1B). These findings are in agreement with another study which reported IAV partitioning to settled solids in wastewater^20^. Based on our findings, primary sludge and municipal wastewater sample processing and viral signal measurements for IAV were performed using solids-optimized enrichment and extraction methods.

### Surveillance of wastewater IAV & IBV RNA citywide 

**Figure 2:**
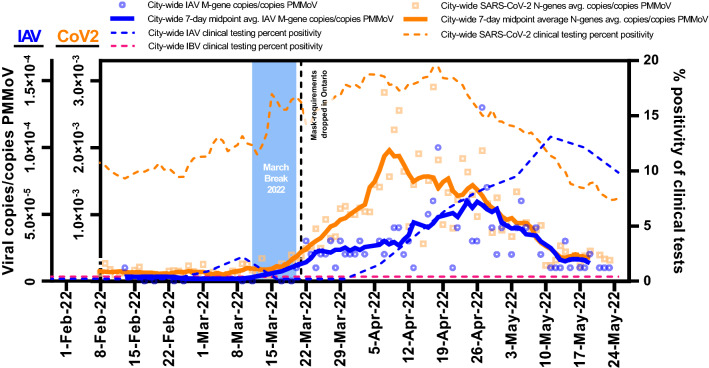
A comparison of both IAV and SARS-CoV-2 wastewater signals citywide in Ottawa, from samples harvested from the city’s WRRF. It was demonstrated that the detection of IAV in wastewater at the city-level (Feb. 13, 2022) occurred 17 days before the first clinical detection of IAV at any of the city’s hospitals or clinics.

The City of Ottawa’s unseasonal influenza A outbreak in the spring of 2022 corresponded with a SARS-CoV-2 resurgence. These diseases were monitored daily from February 1st, 2022, until March 24, 2022 through WWS. IAV was detected in 79 (60%) of the 131 samples tested, while IBV was not detected. Meanwhile, SARS-CoV-2 viral signal above the limit of quantification^22^ was detected in 123 out of the 131 samples tested (94%). IAV and SARS-CoV-2 signals were normalized using PMMoV signal to account for human fecal content in the wastewater solids (Fig. 2). PMMoV is recommended as a normalizing factor to enable identification of trends due to its stability in wastewater and its low temporal variation in concentration, particularly when analyzing the solid fraction of wastewater^6,23–29^.

Mandated masking requirements in the Province of Ontario, which were applied during the COVID-19 pandemic, were largely lifted on March 21, 2022. Lifting of the mandate combined with a one-week student March break (ending March 21, 2022) coincided with a predictable increase in the seven-day average PMMoV-normalized concentration of SARS-CoV-2 along with the initiation of an IAV wave in Ottawa (Fig. 2). The citywide IAV signal did not increase as markedly as that of SARS-CoV-2 during the same post-masking mandate resurgence. Distinction between the rates of increase of IAV and SARS-CoV-2 signal in the city can likely be attributed to factors such as population susceptibility to the diseases, disease transmissibility, incubation period, and possible differences in degradation rates of the disease targets within the sewer system and within primary sludge clarifiers. IAV typically has a shorter mean incubation period (2.0 days)^30–32^ compared to ancestral SARS-CoV-2 (6.4 days)^33,34^, parainfluenza (4.0 days)^32^, respiratory syncytial virus (5.0 days)^32^, SARS-CoV-2 Delta variant (4.8 days)^35,36^ and Omicron variant (3.6 days)^35,36^. Interestingly, the decline of both wastewater viral signals was observed at a similar rate and ended on similar dates. This similarity may simply be due to the end of the flu season coinciding with the exhaustion of Omicron BA.1 in the city. Alternatively, population-wide behaviour may have imparted a similar impact on the transmission of both viruses. Regardless of which hypothesis is correct, the observed overlap of the virus wastewater profiles the of viral interference, whereby the presence of one virus diminishes the spread of another^37^.

Citywide PMMoV-normalized IAV concentrations in primary sludge showed moderate correlation with weekly IAV clinical positivity rates (Pearson’s r = 0.50, *p* < 0.05, n = 14). During the study period, an average of 184 ± 62 weekly clinical Influenza tests were performed in Ottawa, with a maximum weekly test rate of 312 performed in the first week of May. This moderate correlation was observed without the application of a time-step shift being applied to the IAV wastewater signal, which precedes the clinical cases. Time-step correlation analysis revealed that when shifting the IAV wastewater signal 17 days forward, the Person’s R correlation coefficient between the wastewater signal and clinical data increased remarkably (r = 0.97, *p* < 0.05, n = 14). The 17 day overall lead time of the IAV wastewater signal had the highest Person’s R correlation compared to time-steps tested over the range 0–21 days. In this study, the lead time of wastewater surveillance compared to clinical surveillance is the time difference between the identified progression of the outbreak measured in wastewater versus its identified progression by clinical surveillance. The specific lead times of WWS over clinical testing related to initial detection in the wastewater, outbreak detection, peak, and resolution, ranged from 15 to 21 days (Table 1).

**Table 1:** Lead time of normalized IAV viral RNA signal to weekly IAV percent positivity.

Outbreak progression	Lead time of wastewater to clinical metric	Details
First detection	17 days	WWS: IAV in population first identified Clinical: IAV in population first identified
Outbreak detection	21 days	WWS: First 3 consecutive days of increasing IAV viral signal Clinical: First 2 consecutive weeks of increasing weekly percent positivity
Peak of outbreak	15 days	WWS: Date of highest 7-day midpoint avg. IAV viral signal Clinical: Date of highest percent positivity
Outbreak resolution	17 days	WWS: 7 consecutive days of decreasing 7-day midpoint avg. IAV viral signal Clinical: 2 consecutive weeks of decreasing weekly percent positivity

Several factors can explain the extensive lead time for WWS. First, IAV clinical testing in Ottawa is regulated by Public Health Ontario and is typically prescribed when patients meet one of four conditions: (i) pediatric emergency room patient (< 18 years of age) with respiratory infection symptoms, (ii) hospitalized inpatient showing signs and symptoms of respiratory infection, (iii) patient showing signs and symptoms of respiratory infection in an institution where an outbreak has been declared, and (iv) patient showing signs and symptoms of respiratory infection in an institution where an outbreak has not been declared^38^.Constraints imposed on clinical testing access, such as those listed above, cause underestimation of the prevalence of the disease compared to random sampling^39^. Since WWS is independent of clinical testing, it is not affected by such constraints and provides an unbiased representation of the temporal prevalence of the disease in this regard^40^. Hence, WWS of SARS-CoV-2 may provide similar predictive power to that of random clinical testing, and we hypothesize that the same holds true for influenza WWS^41^. Second, clinical test data is reported citywide by the date of clinical testing and not by the onset of symptoms, making it likely that fecal shedding of viral RNA could begin before individuals seek medical treatment and are clinically tested^42,43^. Lastly, due to the well-documented spread of many diseases in younger children at early-childcare centers and schools^44–47^, IAV likely infects large numbers of individuals (young children and their parents) and is relatively undetected by current clinical surveillance before it transitions to more vulnerable populations (e.g., the elderly, sick or immunocompromised) who may find themselves in greater need of medical treatment and are more likely to undergo clinical testing.

Although WWS does not predict cases in a population, it is important to highlight that in the current influenza surveillance context in Ottawa, WWS is the surveillance methods which has shown the potential for the earliest identification of influenza activity in the community and to provide a significant early window of time for public health to act. In particular, the 17-day lead time in detecting influenza in the community allows for public health to communicate to its healthcare partners in the city who make clinical decisions on testing, antiviral prescription and hospital staffing. Overall, this demonstrates that WWS more accurately reflects the onset and prevalence of illness in the studied community compared to clinical surveillance and can provide early warning, thereby allowing the implementation of mitigation measures and confirmation of outbreak resolution.

### Surveillance of wastewater IAV & IBV RNA in neighbourhoods 

**Figure 3:**
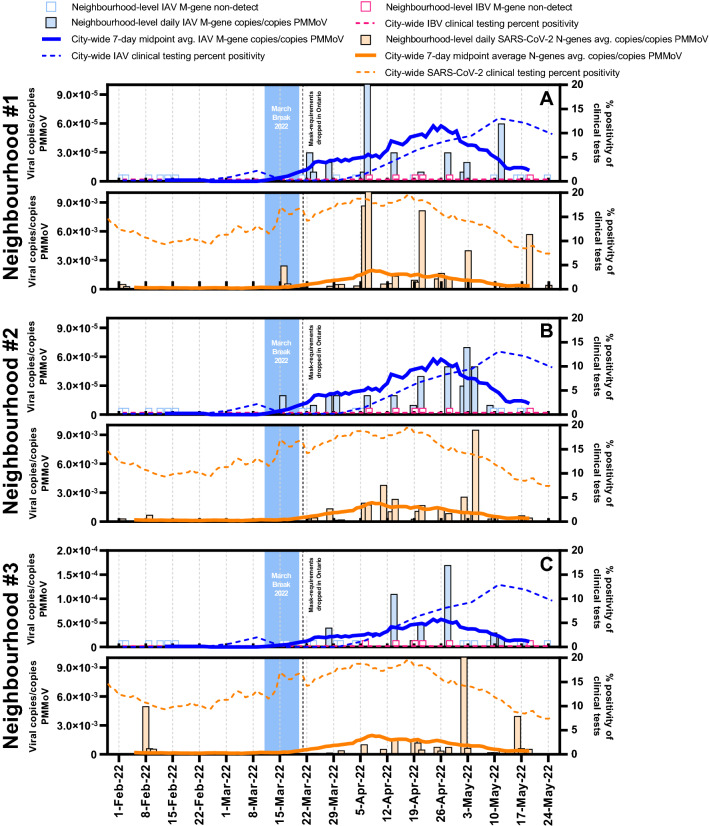
Comparison of both IAV and SARS-CoV-2 wastewater signals at the neighbourhood level (**A**–neighbourhood #1, **B**–neighbourhood #2 and **C**–neighbourhood #3) in Ottawa, for samples harvested from the sewer system. The normalized IAV viral signal differed between neighbourhoods and with the citywide signal when population characteristics differed.

Three neighbourhoods in Ottawa were also monitored for IAV, IBV and SARS-CoV-2 viral signals in wastewater (Fig. 3). Since IBV was not circulating in Ottawa at the time of the study, it was not detected in any of the samples. The neighbourhoods and their sewershed characteristics are described in Table S1. Peaks of both IAV and SARS-CoV-2 viral signals were observed earliest in neighbourhood #1 compared to the other neighbourhoods and the citywide signal. The mean age of residents in this community was significantly lower (approx. 10 years younger) compared to residents in the other two neighbourhoods and the City of Ottawa. We hypothesize that the lower mean age of this neighbourhood indicates the presence of more children, who could have contributed to earlier transmission of IAV and SARS-CoV-2, particularly in the context of lower Covid-19 vaccination rates for young children^48^. Therefore, the wastewater IAV signal peaked in this neighbourhood prior to the other neighbourhoods or within the city as a whole. The early peaking for IAV and not SARS-CoV-2 within the communities may be attributed to distinctions in disease susceptibility and transmissibility in the two cohorts as well as differing sewer infrastructure and associated degradation rates in the associated sewersheds. Neighbourhood #1 saw significantly more elevated viral signals for both IAV and SARS-CoV-2 on several occasions, which supports the idea that more in-community transmission of both viruses occurred during the studied period. The IAV and SARS-CoV-2 viral signals in neighbourhood #2 did not deviate significantly from the citywide signal observed in primary sludge. Coincidentally, this neighbourhood is characterized by population age and number of households that is closest among the three neighbourhoods to citywide averages, perhaps hinting at a similar population distribution. Onset of the IAV outbreak in this neighbourhood was first detected on March 16, 2022, which was after the first detection of clinical cases of IAV citywide. The most elevated PMMoV-normalized IAV viral signal was observed in neighbourhood #3. We believe that because neighbourhood #3 has both the highest population density of all of the neighbourhoods (Table S1), more congregate living situations may exist there, which favors community transmission of IAV^49–51^. Neighbourhood #3 also saw more elevated viral signal levels of SARS-CoV-2 on some occasions, which supports our hypothesis of more in-community transmission of both diseases.

Overall, findings illustrate that detecting both IAV and potentially IBV in sewage samples collected from local, smaller geographical areas may be used as an effective and economical strategy to track the timing, location, and magnitude of influenza activity. However, it has yet to be established if wastewater viral signal differences at the neighbourhood level can be explained by population characteristics, transmission dynamics or a combination of both.

### Subtyping of wastewater IAV RNA citywide and in neighbourhoods 

During the spring of 2022, the City of Ottawa experienced its first outbreak of influenza since the beginning of the COVID-19 pandemic. This outbreak was caused exclusively by IAV, and no IBV viral signal was detected in the wastewater or clinical samples. Therefore, wastewater samples were retrospectively subtyped for H1N1 and H3N2 at a rate of one sample per week. H3N2 was circulating IAV subtype in the three neighbourhoods and citywide, while H1N1 was not detected. H3N2 was identified citywide in the primary sludge via RT-qPCR on February 13, 2022, 17 days earlier than detection of the first case of IAV by clinical surveillance or subsequent clinical subtyping of the virus. In Eastern Ontario, only two out of the four eligible sample categories, samples collected from the first four patients of an institution with a respiratory illness outbreak and samples from patients in institutions not experiencing an outbreak, are sent to Public Health Ontario Laboratories (PHOL) where they are typed and subtyped for H1 and H3. At the peak of influenza testing in Ottawa in 2022, these samples represented only 18% of all testing. The remaining 82% corresponded to samples collected from symptomatic hospitalized patients or symptomatic children who had been admitted to the ER, which were sent to the Eastern Ontario Regional Lab Association (EROLA) where they were only typed for IAV or IBV. The majority of the clinical tests performed were not subtyped and thus important information was absent from clinical testing regarding the state of the circulating subtypes, which can fluctuate during seasonal outbreaks^52^. IAV subtyping via RT-qPCR WWS is nearly real-time and uses minimal resources and infrastructure, which creates unique opportunities for use as and additional tool for public health units/agencies when they carry out clinical surveillance.

## Conclusion

Public health agencies use clinical data such as outpatient visits, hospitalizations, and clinical test results to conduct influenza surveillance, and this data reflects the test date rather than the date of disease onset. Consequently, a lag exists between the community spread of influenza and detection of increased prevalence using traditional approaches. In addition, the clinical data does not accurately indicate the beginning, peak, and end of a given influenza outbreak, nor does it accurately capture the magnitude of influenza illnesses in the community given that most milder sickness, which account for the majority of cases, go undetected.

Our results show that IAV could be quantitated and subtyped through WWS 17 days prior to detection by traditional clinical surveillance. Detecting both IAV and potentially IBV in sewage samples could be a strategy for effective surveillance of influenza viruses in terms of date of emergence, location, and magnitude of outbreaks. By accurately reflecting the whole population burden and onset of illness, WWS serves as an invaluable adjunct to clinical surveillance, informing the implementation of appropriate public health and patient management strategies.

## Methods

### Site descriptions

Wastewater samples collected in this study were harvested from the City of Ottawa’s Robert O. Pickard Environmental Centre, which is the city’s sole water resource recovery facility (WRRF) and processes wastewater from approximately 910,000 individuals or approximately 91% of the Ottawa’s population. In addition, wastewater samples were harvested from three manholes that enabled access to city sewer collectors. These locations geographically isolated three distinct city neighbourhoods that were previously identified as communities that are vulnerable to COVID-19 due to their higher population densities and higher percentages of residents working in congregate care settings (e.g., long-term care facilities). Further information about the sampling locations is provided in Table S1.

### Wastewater sample collection, enrichment, and nucleic acid extraction

To obtain samples that were representative of the citywide viral load of IAV and IBV, hourly 24-h composite samples of primary clarified sludge were collected daily from the Ottawa WRRF between February 2, 2022, and May 24, 2022. For samples representative of neighbourhood-level viral loads of IAV and IBV, hourly 24-h composite municipal sewer wastewater samples were harvested two to three times a week from the neighbourhoods across the same period. Upon collection, the primary sludge samples were immediately refrigerated at 4 °C at the WRRF to await transport, and they were placed in coolers with ice packs for transport to the laboratory for analysis. Harvested neighbourhood samples were immediately placed in coolers with ice packs and transported to the laboratory, where they were allowed to settle for 60 min followed by decanting to obtain settled solids. Forty milliliters of well-homogenized primary sludge (WRRF samples) or 40 mL of settled solids (neighbourhood sewer samples) was centrifuged and 250 mg of the pelleted material was processed using the RNeasy PowerMicrobiome (Qiagen) methodology as previously described by D’Aoust et al.^22^.

### Wastewater sample RT-qPCR analysis

Measurements of IAV, IBV, pepper mild mottle virus (PMMoV), and SARS-CoV-2 were performed via singleplex RT-qPCR (Bio-Rad, Hercules, CA) using previously developed assays^23,53–55^. All primers and probes, PCR cycling conditions, and reagent concentrations are described in Table S3. Each sample was run in triplicate with non-template controls and five-point standard curves prepared with an RT-ddPCR-quantified lab-propagated Hong Kong/1/68/MA/E2 (H3N2) strain of IAV (IAV positive control), and the EDX RPPOS standard (Exact Diagnostics) (IBV positive control). The assay’s limit of detection (ALOD) and quantification (ALOQ) for IAV’s M gene region were approximately 3.5 and 5.7 copies/reaction, respectively. PCR efficiency ranged from 91–102% and R^2^ values were greater than 0.98 (n = 10).

### Primary sludge and municipal wastewater fractionation experiments

Primary sludge collected from the City of Ottawa and municipal wastewater collected from three Ottawa neighbourhoods were used to determine the fractionation of endogenous influenza RNA in these two wastewater matrices. As this study was performed during an outbreak of IAV activity in the City of Ottawa during the spring of 2022, the fractionation experiments were performed on IAV positive primary sludge and municipal wastewater samples. All replicates were subject to the same storage, transport, and holding times prior to the fractioning experiments. Each fraction was extracted within two hours of performing the fractionation procedure.

**Figure 4:**
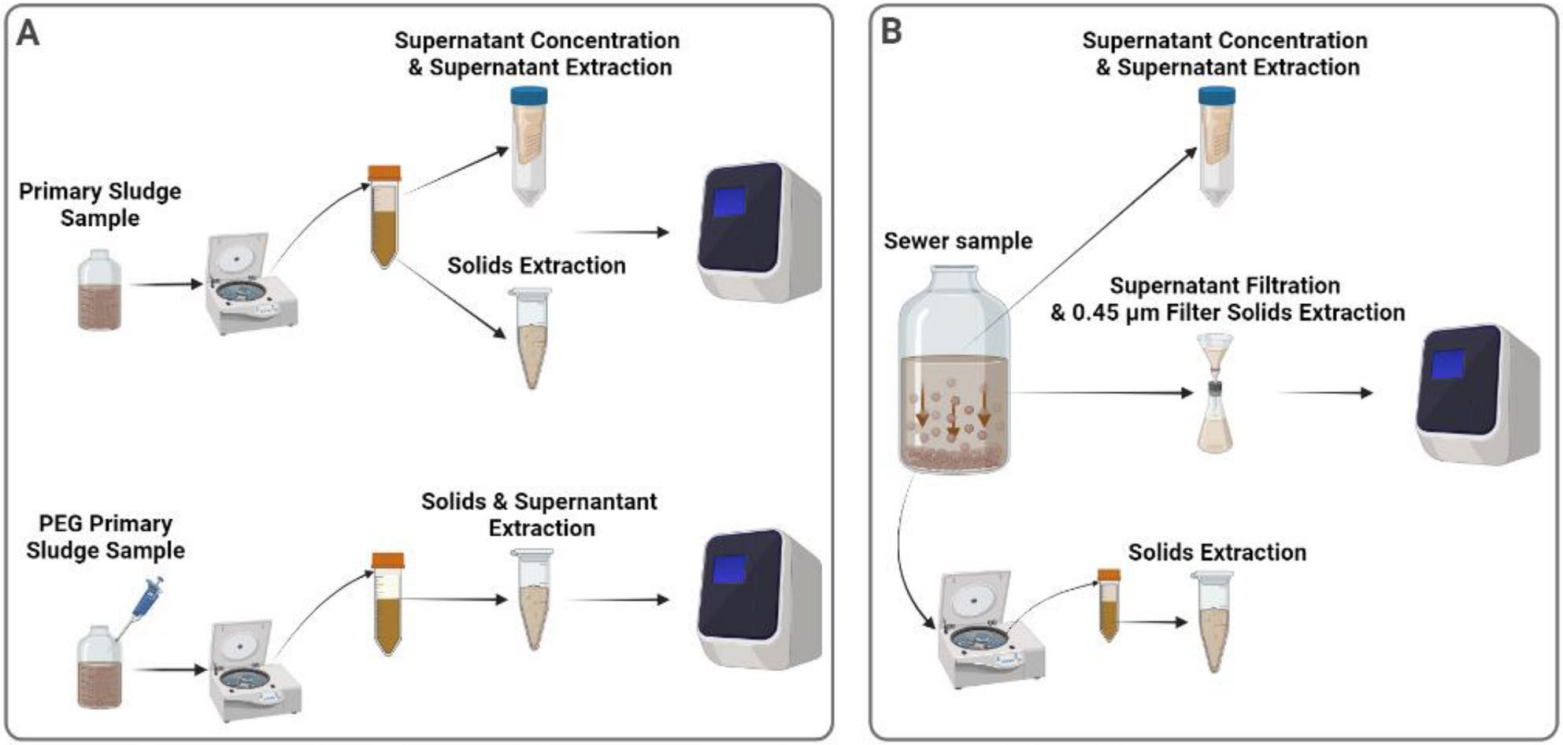
Process flowchart describing: (**A**) Processing of primary clarified sludge samples to examine the fractionation of influenza A viral signal within the supernatant and solid pellet with and without PEG addition, and (**B**) Processing of municipal wastewater to examine the fractionation of influenza A viral signal within the supernatant, filtered suspended solids and solid pellet.

Two 250 mL primary sludge samples were collected on different days and subsequently split into three biological replicates (n = 6) and separated into three fractions: settled solids, PEG-precipitated solids, and supernatant (Fig. 4A). To study primary sludge fractioning, samples were completely mixed, and 30 mL of sludge was collected and centrifuged at 10,000 × g for 45 min at 4 °C. The supernatant was decanted and set aside with care so as to not disturb the pellet. Samples were again centrifuged (10,000 × g, 10 min, 4 °C) and the remaining supernatant was decanted and set aside. The remaining pellet was then stored at 4 °C until extraction. The pellet was considered to be the settled solid fraction of the sample, and it was processed using the RNeasy PowerMicrobiome Kit (Qiagen) as previously described by D’Aoust et al*.*^6^. The supernatant (~ 27 mL) was serially filtered through a 30 kDa–15 mL Amicon ultrafiltration cartridge (EMD Millipore) at 4000 × g for 15 min at 4 °C. The supernatant was then processed using the QIAamp Viral RNA Mini Kit (Qiagen) on a QIAcube Connect automated extraction platform as per the manufacturer’s instructions. Finally, 30 mL of sludge was treated with a PEG 8000 solution at a final concentration of 80 g/L, 0.3 M NaCl, and its pH was adjusted to 7.3 in a final volume of 40 mL. The samples were then mixed and incubated overnight at 4 °C. Each sample was then centrifuged at 10,000 × g for 45 min at 4 °C, and then centrifuged again at 10,000 × g for 10 min at 4 °C. The remaining pellet was then stored at 4 °C until further processing. The sample fraction was considered to be the PEG-precipitated solids fraction of the sample, and it was processed using the RNeasy PowerMicrobiome Kit (Qiagen) following methodologies previously described by D’Aoust et al*.*^22^.

To determine the fractionation of endogenous IAV in municipal wastewater, three 4.0 L of municipal wastewater was collected on different days from the three neighbourhoods in Ottawa, split into four biological replicates (n = 12), and separated into three fractions: settled solids, 0.45 µm filter solids, and supernatant (Fig. 4B). For municipal wastewater fractioning, samples were settled for two hours at 4 °C and then 40 mL of the settled solids were harvested and centrifuged at 10,000 × g for 45 min at 4 °C. The supernatant was decanted and set aside, being careful to not disturb the pellet. Samples were recentrifuged at 10,000 × g for 10 min at 4 °C and the remaining supernatant was again set aside. The final pellet, which was considered the settled solid fraction, was stored at 4 °C until extraction and processed using the RNeasy PowerMicrobiome Kit (Qiagen) as previously described by D’Aoust et al*.*^6^. Subsequently, 40 mL of the post-settled supernatant of each sample was serially filtered through a 30 kDa–15 mL Amicon cartridge at 4000 × g for 15 min at 4 °C to generate the supernatant fraction. This was processed using a QIAamp Viral RNA Mini Kit (Qiagen) on a QIAcube Connect automated extraction platform as per the manufacturer’s instructions. Finally, 500 mL of supernatant (post-settling) from each sample was serially filtered through a 1.5 µm glass fiber filter (GFF) followed by filtration through a 0.45 µm GF6 mixed cellulose ester (MCE) filter (EMD Millipore). Thirty-two milliliters of elution buffer (0.05 M KH2PO4, 1.0 M NaCl, 0.1% (v/v) TritonX-100, pH 9.2) was then passed through the spent filter (1.5 µm and 0.45 µm). The resulting eluate, which was considered to be the 0.45 µm filter solids fraction, was captured and stored at 4 °C until processing using an RNeasy PowerMicrobiome Kit (Qiagen) as previously described by D’Aoust et al*.*^6^.

Several enrichment methods were used in this study to partition the primary sludge and municipal wastewaters. In particular, we used PEG precipitation, filtration, and centrifugation, as these methods satisfactorily concentrate enveloped viruses in wastewater^19,56–65^. In addition, two RNA extraction kits were used to analyze the distinct solids and liquid fractions of the primary sludge and municipal wastewater samples. The RNeasy PowerMicrobiome Kit (Qiagen) extraction kit was used to process solids-rich fractions and has been previously demonstrated to preferentially extract viral RNA from solids. The QIAamp Viral RNA Mini Kit (Qiagen) extraction kit was used to process liquid fractions and has been demonstrated to preferentially extract viral RIN from the liquid fraction of wastewaters^54,66–68^.

The percentage of IVA gene copies partitioned to the primary sludge solids fraction was calculated by multiplying the gene copies measured in the extracted mass of solids by the ratio of the total mass of pelleted solids to the extracted mass. PEG precipitation was applied to the primary solids to calculate the viral signal present in the unsettled/suspended solids of the liquid fraction that was not detected by analyzing the liquid fraction alone. Thus, the IAV viral signal in the PEG-precipitated solids fraction was calculated by subtracting the IVA gene copies measured in the solids precipitated via PEG from the IVA gene copies measured in the primary sludge solids fraction without PEG. Finally, IVA gene copies in the primary sludge supernatant fraction were calculated by multiplying the gene copies measured in the extracted liquid phase by the ratio of the total liquid volume to the extracted volume. The total measurable endogenous IAV signal from the primary sludge samples was subsequently calculated by summing the IVA gene copy fractions. Partitioning of the total signal was expressed as the percentage of the total measured signal of the fractions.

The number of IVA gene copies in the municipal wastewater solids fraction was calculated by multiplying the gene copies measured in the extracted mass of the solids by the ratio of the total mass of pelleted solids to the extracted mass. The IVA gene copies in the 0.45 µm filtered solids fraction and supernatant fraction was calculated by multiplying the gene copies measured in the extracted sample by the ratio of the total sample volume to extracted volume. The total measurable endogenous IAV signal from the municipal wastewater samples was calculated by adding the three IVA gene copy fractions together. Partitioning of the total signal was expressed as a percentage of the total measured signal.

### Clinical data

All influenza clinical data was provided weekly by Ottawa Public Health. Influenza screening of patient clinical samples in Ottawa was performed via RT-PCR assays either at the regional or provincial level, depending on whether patient samples originated from hospital inpatient testing or institutional outbreaks, respectively. Testing criteria for influenza screening in Ontario included: (i) pediatric (< 18 years old) emergency room patients with respiratory infection symptoms, (ii) all hospitalized inpatients showing signs and symptoms of respiratory infection, (iii) patients showing signs and symptoms of respiratory infection in institutions where an outbreak was declared, and (iv) patients showing signs and symptoms of respiratory infection in institutions where an outbreak was not declared^38^. Types 3 and 4 clinical samples, namely institutional patient samples, were the only ones sent to Public Health Ontario Laboratories (PHOL), where they were typed and subtyped. All other samples were sent to the Eastern Ontario Regional Lab Association (EROLA) to be typed.

### Statistical analyses

Time-step Pearson’s R correlation analyses were performed using Graphpad Prism (version 9.3.1) to evaluate the fit between observed IAV viral signal in wastewater and reported IAV clinical data obtained from Ottawa Public Health, and to determine the lag/prediction period between observed IAV viral signal in wastewater and reported IAV clinical cases at various time steps from zero to 21 days. Normality of the data was established beforehand using a quantile–quantile plot to determine the validity of using Pearson’s R correlation.

### Ethical considerations

Prior to conducting this study, we sought guidance from both the University of Ottawa’s research ethics board and the Canadian research ethics board, and determined that the use of wastewater-acquired viral signals at the neighbourhood level and citywide did not require board review and approval. All clinical data utilized in this study was anonymous and was collected and collated by local public health units in accordance with relevant guidelines and regulations. The use of anonymized clinical data was approved by the ethics committee of Ottawa Public Health. Furthermore, informed consent was obtained from all subjects and/or their legal guardian(s).

## Supplementary Information

Supplementary Information.

## Data Availability

Data is available upon reasonable request by contacting the corresponding author.
